# Repeatability in the contact calling system of Spix's disc-winged bat (*Thyroptera tricolor*)

**DOI:** 10.1098/rsos.140197

**Published:** 2015-01-21

**Authors:** Gloriana Chaverri, Erin H. Gillam

**Affiliations:** 1 Universidad de Costa Rica, Alamedas, Golfito 60701, Costa Rica; 2 Department of Biological Sciences, North Dakota State University, Fargo, ND 58108, USA

**Keywords:** contact call, disc-winged bat, group cohesion, repeatability

## Abstract

Spix's disc-winged bat (*Thyroptera tricolor*) forms cohesive groups despite using an extremely ephemeral roost, partly due to the use of two acoustic signals that help individuals locate roost sites and group members. While the calls that aid in group cohesion are commonly used, some bats rarely or never produce them. Here, we examine whether the differences observed in the contact calling behaviour of *T. tricolor* are repeatable; that is, whether individual differences are consistent. We recorded contact calls of individuals in the field and rates and patterns of vocalization. To determine whether measured variables were consistent within individuals, we estimated repeatability (*R*), which compares within-individual to among-individual variance in behavioural traits. Our results show that repeatability for call variables was moderate but significant, and that repeatability was highest for the average number of calls produced (*R*=0.46–0.49). Our results demonstrate important individual differences in the contact calling behaviour of *T. tricolor*; we discuss how these could be the result of mechanisms such as frequency-dependent selection that favour groups composed of individuals with diverse vocal strategies. Future work should address whether changes in social environment, specifically group membership and social status, affect vocal behaviour.

## 2. Introduction

Studies of behavioural syndromes and animal personality have revealed that, in many taxa, consistent individual differences in behavioural traits are present across contexts and time [1–3]. Understanding individual differences in behavioural traits has the potential to elucidate factors that may pertain to fitness, population dynamics and community-level processes [4–6]. Such research also provides a wealth of evidence that inter-individual variation in behaviour is not noise, but rather an intrinsic characteristic of behavioural systems that warrants further research. However, most research to date on the repeatability of behaviour has focused on studying trends related to boldness, exploratory tendency, activity, aggression and sociability [7], some of which are not easy to define and can be hard to objectively measure in a natural context [8,9].

Behavioural characteristics related to group cohesion, such as the use of contact calls, have important implications for fitness, as being able to easily locate mates, offspring or other group members, and not having to invest time to search for them, can impact reproductive success [10,11]. However, little research has focused on repeatability of these behavioural traits, and only one study to date has addressed the issue of individual consistency in contact calling [12]. Research shows that almost all birds use social calls while foraging to maintain contact with their flock, family or mating partner [13]. Contact calling is also common among social mammals, such as cetaceans [14,15], primates [16], elephants [17,18] and bats [11,19–22]. Given the important role of contact calling during group movements, understanding whether individuals consistently differ in their production of contact calls may provide critical insight into group dynamics.

**Figure 1.**
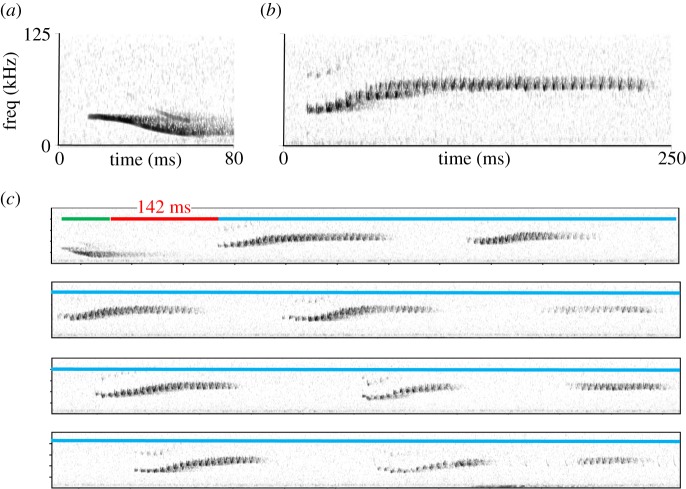
Sonograms showing (*a*) an inquiry and (*b*) a response call used by *Thyroptera tricolor*. (*c*) A bout that includes an emission of an inquiry call (green) and 11 response calls (blue), with time between emission of inquiry call and first response call depicted in red.

One species known to use a complex contact calling system for maintaining group cohesion is Spix's disc-winged bat, *Thyroptera tricolor.* This bat uses an extremely ephemeral roosting resource, the furled leaves of members of the order Zingiberales, which are available for periods ranging from 5 to 31 h [23,24]. Despite constant roost switching, research has shown that *T. tricolor* forms very stable social groups of approximately five to six individuals (range 1–11) whose composition remains unchanged for several years [23,25,26]. Such long-term maintenance of groups is probably aided by a set of vocalizations that help members locate each other during flight and while searching for roosts [27–29]. ‘Inquiry’ calls are simple downward frequency-modulated (FM) sweeps (figure 1*a*) that are emitted by flying bats to maintain contact with flying and roosting group members. ‘Response’ calls are emitted by bats within the roost in response to detection of inquiry calls; these signals are more complex, composed of multiple U-shaped syllables that form a composite signal (figure 1*b*), and are typically emitted in rapid bouts (figure 1*c*). Once response calls are emitted, flying bats rapidly enter the occupied roost [28]. Both call types exhibit individual-specific signatures, although the information capacity of response calls is greater than inquiry calls [30]. In field experiments, results show that flying bats can discriminate among the response calls of roosting bats, preferentially entering leaf roosts from which the calls of group members are being broadcast [29], whereas roosting bats do not preferentially respond to the inquiry calls of flying bats [29]. Given the lower information capacity of inquiry calls [30], coupled with the knowledge that reception of calls through the tubular leaf leads to signal distortion [31], it seems likely that roosting bats are unable to efficiently assess sender identity based on inquiry call structure.

While the two social calls described above are commonly used by *T. tricolor*, some individuals rarely or never produce them [27]. For example, in field experiments where bats were initially captured and then released while recording calls, 66% of individuals tested produced an audible inquiry call. Those that did not produce a call were unable to join group members that were placed inside a tubular leaf [28]. These catch-and-release experiments, along with playback trials, show that bats inside tubular leaves do not all equally respond to detection of inquiry calls; many rapidly and vigorously produce several calls, while some are slower to respond or never do, with concomitant effects on group cohesion [27,28].

Here, we examine whether the differences observed in the contact calling behaviour of *T. tricolor* are consistent within individuals [4]. To estimate consistency, we use repeatability, which is defined as the fraction of variation in a behavioural trait that is attributable to differences between individuals [32,33]. We hypothesize that contact calling behaviour in *T. tricolor* is a repeatable behavioural trait (i.e. within-individual variance is lower than the variance among individuals [33]). We predict that bats will exhibit high repeatability in inquiry call and response call production across recording trials. For response calls, which are only emitted after an inquiry call is emitted, we predict that response call production will also be repeatable despite the identity of the individual producing the inquiry call. In our study, we also assess the potential impacts of sex, age and reproductive condition on calling behaviour, although we had no *a priori* expectations that differences would be present.

We analyse long-term data collected over 3 years on a single population, in which individuals were repeatedly captured and inquiry and response call production was assessed on multiple occasions. While many studies that address repeatability may face difficulties associated with defining and measuring behavioural traits [8], our study addresses a behaviour that is clearly defined and can easily be recorded in a natural context, in addition to being objectively measured. To the best of our knowledge, this is one of the few studies to address the issue of individual consistency in a behavioural trait associated with contact calling (but see [12]) and repeatability associated with acoustic communication [34,35].

## 3. Material and methods

Acoustic data were collected in La Gamba, Piedras Blancas National Park in south-western Costa Rica, from March 2009 to May 2012. Each day we surveyed the area for potential roost sites by locating furled leaves with a diameter of opening ranging between 3.5 and 27 cm, as this is the preferred size range of leaves selected for roosting by *T. tricolor* [24]. Once a roost was located, we captured bats by pinching the top of the leaf and directing them into a cloth holding bag. Bats were fitted with individually numbered metal wing bands, sexed, aged and their reproductive condition assessed. Bats found in the same leaf at the same time were considered a group.

For all individuals and groups captured, we recorded inquiry calls either during free flight or within a small portable flight cage (3×3×2 m). To determine whether bats recorded using both methods vocalized in a similar manner, we applied a General Linear Model in SPSS v. 20 (IBM Corporation, New York, NY, USA). We used as dependent variables the time from trial start to production of first call, and number of calls produced per minute (see methods below for estimating these variables), and as fixed factors bat identity and method of recording (free flight or in flight cage). Only four bats were recorded using these two methods, and we observed no significant differences in their calling behaviour (time of first call: *F*_1,20_=2.18, *p*=0.15; number of calls per minute: *F*_1,20_=0.12, *p*=0.72). Thus, we decided to combine data from both methods into further analyses.

To record inquiry calls, we released a single individual and kept the rest of the group in a waterproof bag to avoid acoustic interference. Within the cage, bats were allowed to fly for a maximum of 3 min; after this period, they were captured using a hand net. Calls emitted by flying bats were recorded with Avisoft condenser microphones (CM16, Avisoft Bioacoustics, Berlin, Germany) through Avisoft's UltraSoundGate 416 or 116 Hm onto a laptop computer running Avisoft-RECORDER software (sampling rate 384 kHz, 16-bit resolution). This process was repeated for all group members. Trials were defined as the period from the start to the end of the bat's flight (typically 3 min in the flight cage) or until the bat was not flying within the recording range (free flight experiments), which was typically less than 1 min.

We performed 216 recording sessions of inquiry calling behaviour on a total of 146 bats. Most bats were sampled only once, but 38 were sampled on two or more occasions, with a maximum of eight trials for one individual. Of the 146 bats, 66 were male and 80 female, but only 14 males and 24 females were sampled repeatedly. A total of 48 sessions were conducted for bats that were juvenile at the time of recording, with the remaining 168 sessions recorded on adults. Only seven young and 29 adult bats were sampled repeatedly, and seven bats were sampled both as juveniles and adults. Among adult females, 24 were non-reproductive, 52 were pregnant and 12 lactating. Time elapsed between the first and last sessions ranged between a few days or months (*n*=18), from 1 to 2 years (*n*=3), 2 to 3 years (*n*=14) and a little over 3 years (*n*=3).

Response call trials began by cutting a suitable tubular leaf and moving it into the flight cage. Because bats only produce response calls after an inquiry call has been emitted [28], we broadcast previously recorded inquiry calls to elicit and record response calls. We band-pass filtered these inquiry calls and broadcast them through an Avisoft UltrasoundGate Player to a broadband loudspeaker (Ultrasonic Omnidirectional Dynamic Speaker Vifa, Avisoft Bioacoustics, Berlin, Germany) to single bats placed inside a furled leaf. To prevent the bats' escape, we placed a circular piece of mesh in the upper portion of the leaf. We placed the microphone near the entrance of the furled leaf, and response calls were recorded onto a laptop computer following the same procedure for recording inquiry calls. Each trial comprises a series of 10 inquiry calls broadcast every 5–10 s. Of the calls selected for these experiments, five were from group members and five were randomly selected among calls from non-group members. We believe this set-up mimicked the social context in which response calling occurs: bats within a roost listen to an inquiry call from an individual flying nearby, either a group or non-group member, and then decides whether to respond or not. We provided mealworms (*Tenebrio molitor*) and water to individuals after each trial and also before releasing them.

A total of 402 recording sessions of response calling behaviour were conducted on 143 bats; 64 were male and 79 female. A total of 53 males and 62 females were sampled repeatedly. At the time of the experiments, 81 sessions were recorded on bats that were juvenile and 321 sessions were conducted on adult bats, while 40 females were non-reproductive, 115 were pregnant and 21 lactating. In total 22 young bats and 97 adult bats were sampled repeatedly, and six bats were sampled both as juveniles and adults. On average, we conducted three sessions per bat, but many individuals were recorded only once, while others were recorded up to eight times. A total of 75 bats were sampled on 2 or more days; among those, 61 were sampled more than once per day. For bats recorded on multiple dates, the time between the first and last response call sessions ranged between a few days (*n*=38) and a little over five months (*n*=9); many sessions were separated by periods ranging between one and four months (*n*=28).

Recordings for both trial types were analysed in SASLab Pro. For each inquiry call trial, we measured three variables: presence/absence of vocal behaviour (inquiry calls), time from trial start to production of first call and number of calls produced per minute. This latter variable was estimated based on the number of calls produced during the time period in which the bat was flying in the flight cage or for the time period in which the bat was observed in free flight trials. For example, if a bat produced one call during 15 s of flight, we estimated that the bat produced a total of four calls per minute. For each response call trial, we measured seven variables: presence/absence of vocal behaviour (response calls), number of calls per bout (average, minimum, maximum and mode) and per cent of inquiry calls that elicited a response in total and after the first response call was produced. We ran a Pearson correlation analysis with the variables number of inquiry calls produced per minute and average number of response calls per bout to determine whether inquiry calling behaviour was correlated to response calling behaviour within individuals.

To determine whether measured variables were consistent within individuals, we estimated repeatability (*R*), i.e. the proportion of the total variance that can be attributed to between-individual variation [36], in R (v. 2.15.2, The R Foundation for Statistical Computing) using the rptr package (http://r-forge.r-project.org/projects/rptr). We used a multiplicative dispersion generalized linear mixed-effects model (GLMM) fitted with the penalized quasi-likelihood (PQL) estimation method, with parametric bootstrapping (*n*=1000) for interval estimation and randomizations (*n*=1000) for significance testing. Analyses were performed on the original and log-transformed data to stabilize variance; for data transformation, we used the log-link (Poisson) and the logit-link (binary) functions. We present results for the link (i.e. transformed) scale and original scale, but interpret repeatability results on the former since these results indicate individual consistency, whereas the latter scale estimates measurement errors [36]. Only bats that were sampled on two or more days were included in the analyses of repeatability.

To determine if sex, age or reproductive status (females only) had an influence on whether bats were vocal or not, we calculated the uncertainty coefficient using the crosstabs procedure in SPSS. This coefficient indicates the proportional reduction in error when values of one variable (in our case the sex, age or reproductive condition of bats) are used to predict values of the other variable (whether bats are vocal or not) [37]. We used a Monte Carlo approximation to calculate significance. We also compared values of two other variables measured for inquiry and response calls to determine whether there were differences between males and females, juveniles and adults, and among females in different reproductive conditions. For this analysis, we used bats that were recorded even for just one session, but only used data from sessions in which bats vocalized. The inquiry call variables we used were (i) time from trial start to production of first call and (ii) number of inquiry calls emitted per minute. For response calls, we used (i) per cent of total inquiry calls that elicited a response and (ii) the average number of response calls produced per bout. Owing to repeated values for several individuals, to evaluate differences among categories we calculated 10 000 bootstrapped standard errors in SPSS. We used two age categories to classify individuals: juveniles and adults. Bats were classified as juveniles if we observed the presence of cartilaginous epiphyseal plates in metacarpals and phalanges [38], if their ventral pelage was white and if there were no signs of reproductive activity, such as testicular descent or an increase in the size of the testes, which is associated with the growth of the seminiferous tubules and spermatogenesis in males, or a bare patch and enlarged keratinized nipples, which indicate parity in females [39]. A combination of all of these latter characteristics occurs in individuals younger than six to seven months old [40]; thus, bats were considered juvenile if they were younger than eight months. Adult females were classified as being pregnant if distension of the female's lower abdomen was present and if a fetus was also palpable; lactating if they had enlarged nipples which upon palpation expressed milk [39]; and non-reproductive if they had an enlarged keratinized nipple but no signs of pregnancy or lactation.

Because response calls are always emitted after an inquiry call, we were interested in determining whether bats would preferentially respond to specific individuals; if this is the case, then we would presume that consistency in response behaviour is wholly or partly explained by identity of the interacting individuals. To determine whether the number of response calls produced by bats was determined by the identity of bats emitting inquiry calls and order in which we presented these calls, we ran a generalized linear model in SPSS. In the model, we used number of response calls as the response variable with a Poisson distribution, and as predictors we used identification of inquiry call and the order in which calls were presented nested within the identity of the bat that was used in the experiment. For these models, we selected a subset of individuals that had responded to inquiry calls at least once, and only included in our analyses those for which we had presented at least two inquiry calls from the same individual. Only seven bats met the previous criteria, and thus we consider our results preliminary.

## 4. Results

For analyses of repeatability, we used data from the 38 and 75 bats that were sampled on two or more occasions for inquiry and response calls, respectively. Overall, repeatability for all variables measured for inquiry and response calls was moderate and significant (range=0.35–0.49 for transformed data and 0.36–0.69 for original data; table 1). For inquiry calls, repeatability was highest for call rate, i.e. the number of calls produced per minute (*R*=0.46), and for whether bats vocalized during flight or not (*R*=0.49). Repeatability for the average number of response calls produced per bout was higher (*R*=0.49) than other repeatability results for response calls. In general, the 95% confidence interval for all variables was large. These results show that within-individual variance is lower than the variance observed among individuals for all calling variables measured, and thus that individuals exhibit consistent differences in their vocal behaviour.

**Table 1.** Summary results for the analysis of repeatability (*R*) for all call variables measured. Results are based on the link (i.e. transformed) and original scales. Confidence intervals (CI) were estimated from parametric bootstrapping, and *p*-values were generated from 1000 randomizations.

		link scale			original scale		
call	variable	*R*	95% CI	*p*-value	*R*	95% CI	*p*-value
inquiry	calls per minute	0.46	0.25–0.68	0.001	0.57	0.27–0.82	0.001
	time of first call	0.35	0.18–0.62	0.01	0.51	0.22–0.84	0.01
	vocalized yes/no	0.49	0.00–0.78	0.01	0.22	0.00–0.40	0.02
response	average no. calls/bout	0.49	0.39–0.70	0.001	0.65	0.44–0.91	0.001
	min no. calls/bout	0.43	0.27–0.57^a^	0.001	0.45	0.23–0.68^a^	0.001
	max no. calls/bout	0.47	0.40–0.74	0.001	0.69	0.48–0.97	0.001
	mode no. calls/bout	0.44	0.36–0.66	0.001	0.57	0.39–0.84	0.001
	per cent responded	0.42	0.36–0.65	0.001	0.66	0.49–0.88	0.001
	per cent responded after first response	0.37	0.32–0.60	0.001	0.60	0.43–0.84	0.001
	vocalized yes/no	0.41	0.18–0.49	0.001	0.36	0.15–0.43	0.001

^a^Estimated overdispersion < 1, thus CI limits are unreliable.

**Figure 2.**
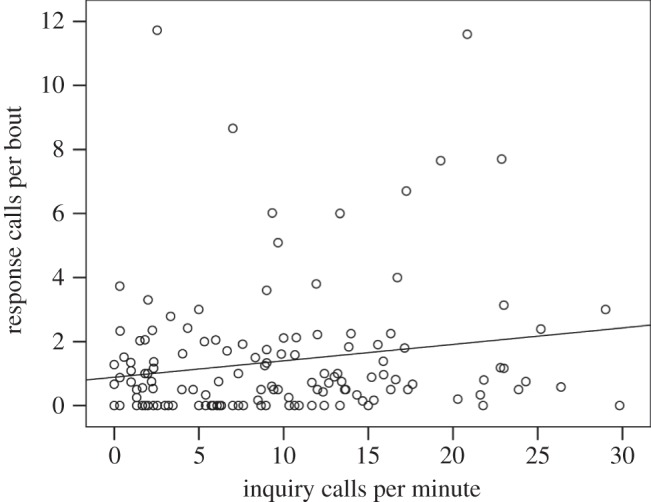
Correlation between the number of inquiry calls produced per minute and average number of response calls.

To determine whether inquiry calling behaviour was correlated to response calling behaviour, we compared the number of inquiry calls produced per minute and average number of response calls per bout for 139 individuals for which we had at least one recording of inquiry and response calls. Our results show that there was a weak, but significant, positive correlation between the number of inquiry and response calls (*r*=0.18, *p*=0.03; figure 2), suggesting that bats which produced more inquiry calls per minute also produced more response calls per bout.

**Figure 3.**
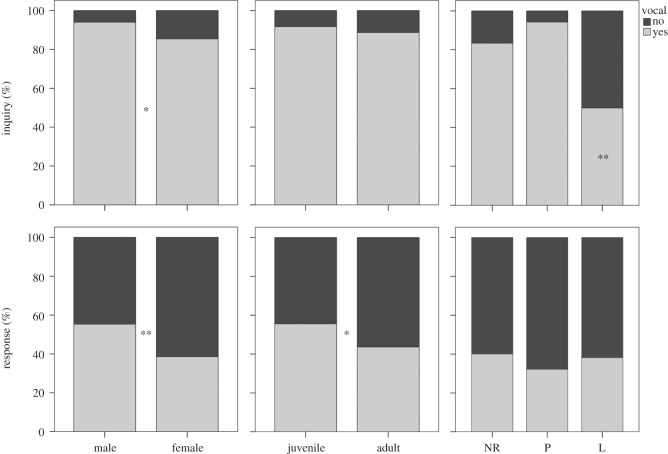
Percentage of times in which bats of different sex, age and reproductive condition vocalized. Asterisks denote significant differences (*p<0.05, ^**^p<0.001) among categories. For inquiry calls, a significant difference was observed in the percentage of sessions in which lactating females (L) vocalized compared to those that were non-reproductive (NR) and pregnant (P).

**Figure 4.**
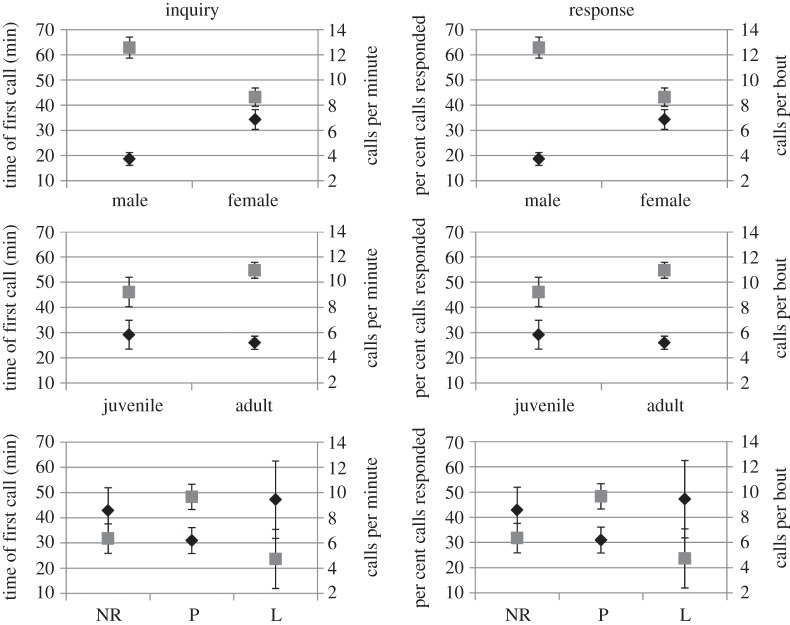
Mean difference in time of first call and calls per minute for inquiry calls, and percentage of calls responded and calls per bout for response calls, between males and females, juveniles and adults, and non-reproductive (NR), pregnant (P) and lactating (L) females. Data for black diamonds correspond to the *y*-axis, and data for grey squares to the *z*-axis. Vertical lines represent the standard error based on 10 000 bootstrap samples.

To assess the potential impacts of sex, age and reproductive condition on calling behaviour, we compared inquiry and response calling variables of 146 and 143 bats, respectively. Males were vocal in a larger proportion of sessions than females for both inquiry and response calls (figure 3). In general, males vocalized sooner and produced more inquiry calls than females (figure 4). However, no differences in the percentage of times that bats responded, nor the number of response calls per bout, were observed between males and females. We found no differences in the proportion of times that juveniles and adults vocalized during flight (figure 3), although juveniles produced fewer inquiry calls per minute than adults (figure 4). We also observed that juveniles produced response calls in a larger proportion of sessions than adults (figure 3), but otherwise were similar to adults in other variables measured for response calls (figure 4). Lactating females produced inquiry calls in significantly fewer sessions than other females (figure 3). In addition, pregnant females were more vocal during flight, producing a larger number of inquiry calls per minute than non-reproductive or lactating females (figure 4). Lactating females, by contrast, were significantly less vocal during playback of inquiry calls compared with non-reproductive females, producing significantly fewer response calls (figure 4). We observed no differences in the proportion of sessions in which a response call was produced by females in different reproductive conditions (figure 3).

**Figure 5.**
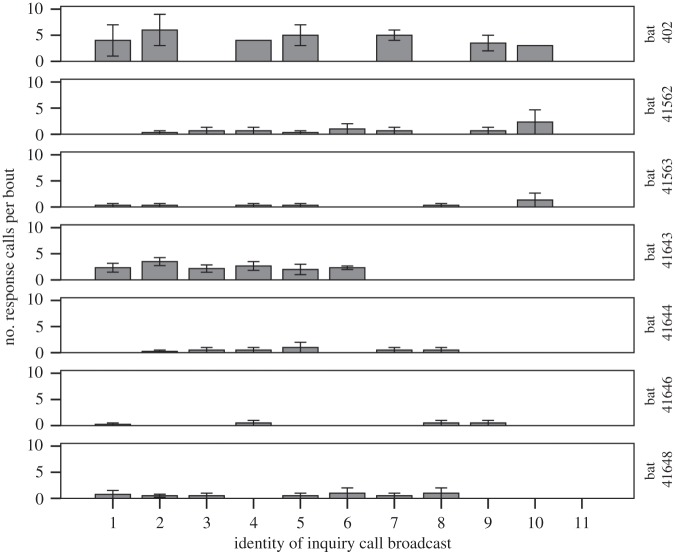
Average number of response calls produced by seven bats according to the identity of the inquiry call that was broadcast. Error bars represent ±1 s.e.

Our generalized linear models to test if bats preferentially respond to specific individuals show that the interaction between the identity of the inquiry call and the order in which calls were presented significantly explains the number of response calls emitted (*χ*^2^=11.44, d.f.=5, *p*=0.04), but each variable by itself was not significant (inquiry call identity: *χ*^2^=4.19, d.f.=8, *p*=0.84; order: *χ*^2^=10.45, d.f.=15, *p*=0.79). Results also confirm that the most important variable explaining the differences in the number of response calls produced was bat identity (*χ*^2^=53.56, d.f.=6, *p*<0.001). These findings suggest that major differences in vocal behaviour persist among bats, but that each individual exhibits preferential responses towards particular inquiry calls and the order in which they are presented. For example, of the seven individuals considered in the analysis, all of them responded to certain, but not all, inquiry calls (figure 5), yet when we compare the average number of calls produced by each individual we can see that bats such as no. 402 and no. 41643 produced many response calls when they vocalized, whereas all other bats produced very few calls.

## 5. Discussion

Contact calling is an important strategy used by social animals to maintain cohesive groups [41]. Few studies to date have determined whether individuals within groups consistently produce contact calls more frequently than others (but see [12]), and whether group movements are controlled by these more vocal members. However, leadership within moving groups provides important clues to understanding the role of particular group members in social cohesiveness [42], with concomitant effects in group performance [43]. Our study on the contact calls employed by Spix's disc-winged bat demonstrates that there are significant differences in the number of contact calls produced among individuals, which may have important implications for understanding group dynamics in this species.

Many hypotheses have been proposed to explain why individuals behave consistently (low intra-individual variation) even when large differences are observed across individuals (high inter-individual variation). One possibility is that groups are more successful when a mix of behavioural strategies is present, especially if such variation means that only some of the individuals in the group incur the risk or energetic cost associated with performing a behaviour [44]. Variance in the production of *T. tricolor* social calls, particularly the response calls used to announce roost location, could be adaptive if only some group members incur the cost of sound production [45], but all benefit from such signalling by rapidly locating a roost site [27,28]. Groups of this species are composed of highly related individuals [46,47], meaning that indirect fitness benefits could also be accrued by individuals that incur the cost of producing response calls [48].

An unresolved question from previous studies on this system is why roosting individuals seem to respond indiscriminately (regardless of whether the inquiry caller is a group member or not [29]), if, as we assume, production of response calls has a significant cost. Preliminary results from our current study suggest that while significant differences in vocal behaviour among bats persist, bats may preferentially respond to specific individuals. Further studies investigating these preferences, the cost of sound production, and the benefits to group performance will help us understand how inter-individual variation in this behaviour is maintained.

Individual consistency in behaviour may arise in response to predictability benefits, such that individuals can reliably infer the future behaviour of group members if all interacting individuals behave consistently [49,50]. There may also be high costs to behavioural flexibility; some of these include expensive maintenance of sensory mechanisms during development and information acquisition costs that individuals must accrue to behave optimally under variable ecological conditions [51]. Both predictability benefits and costs to behavioural plasticity, among other mechanisms for intra-individual consistency [3,52,53], could explain our findings of consistent vocal behaviour in the context of contact calling in *T. tricolor*, yet any conclusions about this topic would be speculative at the moment.

Despite general consistency in vocal behaviour, we also observed some behavioural plasticity. For example, lactating females produced fewer response calls than non-reproductive and pregnant females, which suggests that during times of high energetic expenditure [54], individuals may avoid the additional costs of sound production [45]. However, it remains a puzzle why lactating females produce fewer inquiry calls, as locating suitable roost sites quickly could compensate the energy spent vocalizing. Our results also show that roosting juveniles more readily produced response calls than adults, suggesting that vocal behaviour changes during development. While explanations regarding these differences in vocal behaviour between young and adult bats are still speculative, we propose that juveniles may more readily call if the costs of remaining separated from the group are higher compared with a solo adult. Given that juvenile animals are generally inexperienced hunters [55] that may rely on social learning from adults to improve foraging skills, incurring the cost of increased vocalization may be small compared with the benefit of re-locating adult group members. Why juvenile bats do not produce more inquiry calls during flight, a time when remaining with experienced foragers is more critical, is still a question that deserves further study. Finally, we found that males were more vocal than females, which suggests that the former may be primarily responsible for coordinating group movements during flight and during the location of roost sites. However, these tasks cannot be solely assigned to males because females also commonly vocalized. Other studies have found that males and females produce contact calls in similar proportions (e.g. white-faced capuchins [56]), although most have found that females produce them more frequently than males (e.g. Asian and African elephants, Japanese macaques and De Brazza's monkeys [17,18,57,58]). Unfortunately, the proximate and ultimate causes for these differences in contact call production rates among males and females remain unstudied.

While our results suggest that individual calling behaviour in *T. tricolor* is repeatable, we still do not know whether other factors, such as social status, are related to vocal behaviour. If social status affects vocal behaviour in *T. tricolor*, then the differences we observe in calling rates are not necessarily the product of personalities, but rather the result of current social conditions. To test the hypothesis that social status determines vocal behaviour in *T. tricolor*, it would be necessary to determine whether group switches, which are known to trigger changes in social status in some animals [59], affect this species' vocal behaviour, as has been observed in other taxa [60,61]. Thus, if social status correlates with vocal behaviour in *T. tricolor*, it would be anticipated that changes in group membership would cause changes in vocal roles. Because our data are based on long-term recaptures of individuals, we observed some changes in group membership, but these events were sufficiently rare that analysis could not be conducted. To answer this question, it is necessary to determine first whether any type of social status, role or hierarchy is present in *T. tricolor*, and whether changes in social status result in changes of vocal behaviour over longer time periods and for a larger sample size.

In conclusion, our results demonstrate persistent differences in calling behaviour in *T. tricolor*, a bat that uses an extremely ephemeral roosting resource and heavily relies on acoustic communication for maintaining group cohesion. Whatever the mechanism(s) responsible for the consistent vocal behaviour within individuals, the variability observed between individuals means that groups are most probably composed of individuals with differing degrees of contact call production, which may result in the apparent evolution of social roles or niches (*sensu* [62]). More vocal individuals may provide a significant benefit to overall group fitness, as cohesive groups of animals are known to perform better than less cohesive ones [43]. If group cohesion increases fitness, and if certain individuals are primarily responsible for maintaining cohesive groups, the loss of those individuals may prove highly detrimental to the fitness of groups. Further research is necessary to understand whether groups are indeed composed of a suite of vocal and non-vocal members, whether group cohesion can persist despite loss of the former and whether social roles within groups switch after the loss of these group members.

## Supplementary Material

File “inquiry”: Inquiry call data showing the identification number of the bat tested (Bat), the day (relative to first capture date) in which recording occurred, and the time of first call (in seconds after the bat started flying) and number of calls emitted per minute.

## Supplementary Material

File “response”: Response call data showing the identification number of the bat tested (Bat), the day (relative to first capture date) in which recording occurred, and other measures of calling behaviour (for details see methods section).

## Data accessibility

The datasets supporting this article have been uploaded as part of the electronic supplementary material.
